# Genetic analysis of impaired trimethylamine metabolism using whole exome sequencing

**DOI:** 10.1186/s12881-017-0369-8

**Published:** 2017-02-15

**Authors:** Yiran Guo, Liang-Dar Hwang, Jiankang Li, Jason Eades, Chung Wen Yu, Corrine Mansfield, Alexis Burdick-Will, Xiao Chang, Yulan Chen, Fujiko F. Duke, Jianguo Zhang, Steven Fakharzadeh, Paul Fennessey, Brendan J. Keating, Hui Jiang, Hakon Hakonarson, Danielle R. Reed, George Preti

**Affiliations:** 1 Center for Applied Genomics, the Children’s Hospital of Philadelphia, 3615 Civic Center Blvd, Abramson Res Cntr, Ste 1016H, Philadelphia, PA 19104 USA; 2 Monell Chemical Senses Center, 3500 Market St, Philadelphia, PA 19104 USA; 3 BGI-Shenzhen, Shenzhen, 518083 China; 4 Perelman School of Medicine, University of Pennsylvania, Philadelphia, PA 19104 USA; 5 University of Colorado Health Sciences Center, Denver, CO USA; 6 Shenzhen Key Laboratory of Genomics, Shenzhen, 518083 China; 7 The Guangdong Enterprise Key Laboratory of Human Disease Genomics, Shenzhen, 518083 China; 8 Department of Dermatology, Perelman School of Medicine, University of Pennsylvania, Philadelphia, PA 19104 USA

## Abstract

**Background:**

Trimethylaminuria (TMAU) is a genetic disorder whereby people cannot convert trimethylamine (TMA) to its oxidized form (TMAO), a process that requires the liver enzyme FMO3. Loss-of-function variants in the *FMO3* gene are a known cause of TMAU. In addition to the inability to metabolize TMA precursors like choline, patients often emit a characteristic odor because while TMAO is odorless, TMA has a fishy smell. The Monell Chemical Senses Center is a research institute with a program to evaluate people with odor complaints for TMAU.

**Methods:**

Here we evaluated ten subjects by (1) odor evaluation by a trained sensory panel, (2) analysis of their urine concentration of TMA relative to TMAO before and after choline ingestion, and (3) whole exome sequencing as well as subsequent variant analysis of all ten samples to investigate the genetics of TMAU.

**Results:**

While all subjects reported they often emitted a fish-like odor, none had this malodor during sensory evaluation. However, all were impaired in their ability to produce >90% TMAO/TMA in their urine and thus met the criteria for TMAU. To probe for genetic causes, the exome of each subject was sequenced, and variants were filtered by genes with a known (*FMO3*) or expected effect on TMA metabolism function (other oxidoreductases). We filtered the remaining variants by allele frequency and predicated functional effects. We identified one subject that had a rare loss-of-function *FMO3* variant and six with more common decreased-function variants. In other oxidoreductases genes, five subjects had four novel rare single-nucleotide polymorphisms as well as one rare insertion/deletion. Novel in this context means no investigators have previously linked these variants to TMAU although they are in dbSNP.

**Conclusions:**

Thus, variants in genes other than *FMO3* may cause TMAU and the genetic variants identified here serve as a starting point for future studies of impaired TMA metabolism.

**Electronic supplementary material:**

The online version of this article (doi:10.1186/s12881-017-0369-8) contains supplementary material, which is available to authorized users.

## Backgrounds

The human body produces many chemicals that have an odor. Sometimes these odors arise through normal cellular and metabolic processes, but sometimes they arise from inborn errors of metabolism. One example of an odorous chemical the body produces is trimethylamine (TMA), which has the unpleasant odor of fish [1]. TMA is derived from dietary choline and similar compounds (e.g., carnitine, betaine, taurine) found in many foods. When choline is ingested, the gut bacteria cleave the TMA moiety from it [2]. The free TMA is oxygenated by the human liver enzyme flavin containing monooxygenase 3 (FMO3), a member of the oxidoreductase class of enzymes, to the odorless TMA-N-oxide (TMAO). Thus, under normal circumstances, choline ingestion does not result in body odor through this pathway. However, people who have genetic variants of the *FMO3* gene that inactivate or lower its efficacy secrete the odorous TMA in urine, sweat and other body fluids [3, 4].

Impaired trimethylamine metabolism is assessed by tracking the ratio of TMAO to TMA and in the urine after the person ingests a fixed amount of choline, a challenge test [5]. While the syndrome is best known for its fish-like body odor, in our experience, the odor is episodic and may not be detectible at a single time point [6] although oral odor, unrelated to TMA metabolism (e.g., chronic halitosis) is common in these subjects as it is in the general population [7]. The fish-like odor may not be present during an assessment due to recent diet (e.g., little or no choline) or other vicissitudes of life, e.g., [8–10]. Two other possibilities are 1) the transient form of TMAU in children [11, 12], and 2) the odor might be present but not detected because the assessor cannot smell TMA [13] due to a specific anosmia in the human population.

TMAU is a genetic disorder. Specifically variants in the FMO3 gene are related to impaired trimethylamine metabolism [3, 4, 14–19]. However, some people with TMAU as defined by the choline challenge test do not have any variants, either rare ones with total loss of function or common ones (associated with less extreme impairment) in this gene. Thus, variants in other genes could contribute to this disorder, especially those genes and their protein products within the pathway that process trimethylamine in the same or a similar manner as the FMO3 enzyme. At least two lines of evidence support this hypothesis. Variants of the *DMGDH* gene may lead to a syndrome whereby subjects have a similar body odor complaint (dimethylglycine dehydrogenase deficiency; OMIM #605850, [20]) although the links between odor and this genetic syndrome are speculative. In a second line of evidence, variants in the pyridine nucleotide-disulfide oxidoreductase domain 2 (*PYROXD2*) gene is associated with the concentration of TMA in the urine of healthy people [21, 22]. To determine whether variants in novel genes contribute to this disorder, we evaluated TMA metabolism by choline challenge, conducted a sensory evaluation and sequenced the exome of ten subjects with complaint of TMA-related body odor.

Whole exome sequencing (WES) examines protein-coding regions of the entire human genome to identify genetic variants, including single-nucleotide polymorphisms (SNPs), and small nucleotide insertions/deletions of single or multiple nucleotides (indels). The amount of genetic variation among all people is large, and separating benign from potentially pathological variation is complex [23]. Here we used three criteria to find variants of interest. First, we looked for variations in genes previously associated with impaired TMA metabolism, e.g., the FMO3 gene. Second, we looked for the presence of a potentially rare and pathogenic variant in other oxidoreductases with functions similar to those of FMO3; and third, we evaluated the *in silico* pathogenicity of each variant, its allele frequency, and its presence shared by at least two subjects. We reasoned that a rare variant with potential functional significance observed in two or more subjects was more likely to impair TMA metabolism. These three criteria together helped to reduce the large set of observed variations to a manageable number worthy of further study.

## Methods

### Subject recruitment

We informed adult subjects with body odor complaints who contacted the study investigators about participating in our ongoing sensory and genetic studies. Approximately 130 subjects made contact during an 8-year period from 1999 to 2007 and we evaluated them for the sensory and metabolic arms of the study. From this pool of subjects, we chose ten at random for WES.

### Choline challenge

For the choline challenge test, we instructed subjects to fast from 10 pm the night before testing and collect their first morning urine while at home. This collection was done using a large, plastic container provided in advance; as previously described [5]. The container was pre-loaded with 2 ml of 6 N HCl to convert free TMA to its non-volatile hydrochloride salt. After the subjects arrived in the lab and following the recording of first sensory measures as described below, they ingested 5 g choline dissolved in orange juice. Each subject took three containers home and collected urine over the next 24 h, using one container for each 8-h period. Subjects returned the following day with their urine. The volume in each container was recorded and a 100 ml aliquot removed to a smaller plastic container and frozen. These urine samples were shipped on dry ice and assayed for TMA and TMAO at the University of Colorado Health Sciences Center following previously published procedures [5], with steps for standard solution preparation, water/urine test solution preparation for TMA analysis, and choline loading analysis. We quantified the amount of TMA for each 8-h sample; we measured the sample with the highest concentration of TMA for TMAO. We used the ratio of TMAO to TMA as the key outcome measure: normal reference ranges are ≥0.90.

### Sensory panel evaluation

For the sensory evaluation of body odor, subjects were asked to come to the laboratory fasted and without having brushed their teeth after 10 pm (22:00) the night before; in addition, subjects were asked to refrain from having applied any scented products (e.g., cosmetics and deodorant products) for 3 days prior to testing. Upon arrival at the laboratory, two trained sensory judges evaluated their breath, axillary and upper body odor with quality descriptors (e.g., fishy or sulfurous) using previously described procedures [7]. We repeated these ratings the following morning, after the choline challenge test.

### Genomic DNA

When subjects returned their urine to the laboratory, they gave a 20 ml sample of whole blood by venipuncture. DNA was extracted (QIAamp DNA Mini Kit; Qiagen, Valencia, CA, USA), quantified by spectrophotometry (Nanodrop, Wilmington, DE, USA), and used in the exome analysis and for other follow-up genotyping.

### WES and variant calling

Genomic DNA of 10ug each from ten subjects was part of a large scale WES project of rare genetic diseases between the Center for Applied Genomics at the Children’s Hospital of Philadelphia and BGI. Exomes were captured using the Agilent SureSelect Human All Exon Kit V2 (Agilent Technologies, Santa Clara, CA, USA) and sequenced using the HiSeq 2000 machine (Illumina, San Diego, CA, USA) with standard paired-end sequencing protocol. We conducted subsequent bioinformatics as previously described [24]. Briefly put, raw sequencing reads were stored as FASTQ files and then aligned to the human reference genome (UCSC hg19) with the Burrows-Wheeler alignment [25]. Variant calling was performed using the Genome Analysis Tool Kit (version 1.4) [26] followed by functional annotation using Annovar [27] and SnpEff [28]. We identified all variant sites in a larger set of exomes, and genotypes for any location with one or more minor alleles were determined for all subjects. Variant calling was also performed independently at BGI as previously described [29], and high concordance of results was obtained. For pathogenicity, each variant was assigned two scores using algorithms implemented in Phenotyping version 2 (PolyPhen-2) and Sorting Intolerant From Tolerant (SIFT) [30, 31], respectively. Genes whose products have oxidoreductase activity or that are in the same pathway as FMO3 were identified through a Gene Ontology website (*N* = 729) [32]. We also identified variants where two or more subjects had the same rare minor allele. The final filter was for the minor allele frequency (MAF) <0.05 as reported in the 1000 Genomes data set [33]. We identified variants that met the criteria for potential pathogenicity and rarity, and shared by two or more subjects.

### Gene network analysis

Harnessing gene-gene/protein-protein network relationships, we generated a list of genes that may interact with *FMO3, PYROXD2 and DMGDH,* and investigated rare deleterious variants within these genes. The interacting partners were collected from the STRING database [34] with default parameters below, setting 1) all possible active interaction sources (including text mining, experiments, databases, co-expression, neighborhood, gene fusion, and co-occurrence); 2) minimum required interaction score of 0.4; 3) max number of interactors to show as 10.

### Two genotyping methods to validate exome sequences

We partially Sanger sequenced DNA from each subjects for the *FMO3* gene and conducted Taqman genotyping of the *FMO3* and *PYROXD2* variants to gauge WES reliability. For Sanger sequencing, Exons 2–9 of the *FMO3* gene were amplified by validated primers [3] and submitted for analysis to the DNA Sequencing Facility at the University of Pennsylvania Perelman School of Medicine. Sequencing reactions were performed in an ABI GeneAmp 9700 thermal cycler, resolved with an ABI 3730 DNA sequencer, and analyzed using ABI Sequencing Analysis software, version 5.1 (Applied Biosystems, Foster City, CA, USA). Sequences were aligned using the algorithms implemented in Sequencher (Gene Codes Corporation, Ann Arbor, MI, USA), and the variants were identified [35] and matched against those obtained by WES. We used Taqman methods to type four specific variants, three in the *FMO3* gene (rs2266780, rs2266782, and rs2066532) and one in the *PYROXD2* gene (rs7072216). The *PYROXD2* variant is located in the intronic region thus not captured by WES, but it was reported in a previous paper for significant association with TMA in urine [21]. Appropriate primers and probes were cycled (StepOnePlus, Life Technologies, Grand Island, NY), and variants were called using previously described methods [36].

## Results

### Odor evaluation and trimethylamine metabolism

As summarized in Table 1, of the ten subjects studied here, eight were female and two were male. Six subjects reported their race as Caucasian, and four as African American. Table 1 also summarizes subject demographics and experimental outcomes. All subjects produced abnormally low amounts of TMAO from choline and met the criterion for TMAU, but the TMAO:TMA ratios ranged widely, from 0.13 to 0.87 (normative values >0.90). However, the sensory panel judged most subjects to have unremarkable body odors, with one exception: subject 98 had musty and damp odor. No subjects at the time of evaluation had a fishy odor.

**Table 1:** Characteristics of subjects with body odor complaints

ID	Age (yr)	Sex	Race	TMAO: TMA^a^	Fishy body odor	Fishy oral odor	Other, body odor	Common oral odors
52	78	F	C	0.13	None	None	None	Pungent, sulfurous^b^
114	18	M	AA	0.37	None	None	None	Sulfurous/fecal^b^
122	64	F	AA	0.47	None	None	None	Fecal, pungent^b^
35	45	F	C	0.54	None	None	None	Mild sulfurous^b^
99	59	F	AA	0.58	None	None	None	Mild metallic, smoky
64	51	F	C	0.61	None	None	None	Mild
62	51	F	AA	0.79	None	None	None	Sulfurous^b^
113	47	M	C	0.79	None	None	None	Strong sulfurous^b^
98	44	F	C	0.86	None	None	Musty^c^	Unremarkable
56	70	F	C	0.87	None	None	None	Unremarkable

*ID* subject identifier number, *age* age at assessment in years (yr), *M* male, *F* female, *AA* American of African descent, *C* Caucasian American of European descent. Race/ethnicity was determined by self-report. ^a^Table is sorted by TMAO:TMA ratio; Less than 0.90 is criterion for TMAU (i.e., reference > 0.90).^b^Oral odor secondary to plaque located on the posterior dorsal surface of the tongue. Plaque often contains odor-causing bacteria ([7] and references therein). ^c^Also described as ‘damp’

### WES and analysis

From the larger sample set of the parent project (*n* = 669), 817,028 SNP variant sites and 75,781 indel sites were identified (Table 2 and Additional file 1: Table S1). We found seventy percent of these variants in the homozygous state. Due to differences in sequencing coverage missing genotypes rate varied among individuals and accounted for 15 to 23% of SNP variant sites and 25 to 33% of total indel variant sites.

**Table 2:** Filtering of SNPs and indels

Number of variants	SNPs	Indels
Called in entire larger sample (*n* =669)	817,028	75,781
→ Found in all 10 TMAU subjects, with at least one subject homozygous for the minor allele	94,535	5,251
→ Pathogenic by SIFT or PolyPhen-2	1,867	163
→ Pathogenic in oxidoreductase pathways	57	4
→ Pathogenic in oxidoreductase pathways and with MAF < 0.05	4	1
→ Pathogenic in genes with no known choline metabolism function and shared by 2 subjects	762	63
→ Pathogenic rare variant homozygous in two subjects within genes with no known choline metabolism function	9	1

We identified one rare allele within the *FMO3* gene. This subject (#99) was heterozygous for a nonsense variant rs72549325 (chr1:g.171076936G > T; NM_001002294.2:c.442G > T; NP_001002294.1:p.Gly148Ter). We also identified subjects with common variants. Five subjects were heterozygous for rs2266782 (chr1:g.171076966G > A; NM_001002294.2:c.472G > A; NP_001002294.1:p.Glu158Lys), one subject was heterozygous for rs1736557 (chr1:g.171080080G > A; NM_001002294.2:c.769G > A; NP_001002294.1:p.Val257Met) and one subject was heterozygous for rs2266780 (chr1:g.171083242A > G; NM_001002294.2:c.923A > G; NP_001002294.1:p.Glu308Gly). Although common in the population, these three variants of the *FMO3* gene have been associated with impairments in metabolism [37–39]. For the *PYROXD2* gene, the allele of interest is common and we found it in five subjects, three of whom were homozygous. We summarize information about these *FMO3* and *PYROXD2* gene variants in Table 3. In addition, we list 23 more *FMO3* rare gene variants that have role in TMAU in Additional file 1: Table S2. We detected none of these variants in the ten people studied here.

**Table 3:** Known rare and common pathogenic variants for TMAU observed in this study

Gene	Variants (hg19)	MAF	Amino acid change in HGVS format	Subject ID^a^	Ref
*FMO3*	rs72549325 chr1:g.171076936G > A/T NM_001002294.2:c.442G > A/T	4.118e-05/ 8.236e-06^b^	NP_001002294.1:p.Gly148Arg/ NP_001002294.1:p.Gly148Ter	99^†^	[40]
*FMO3*	rs2266782 chr1:g.171076966G > A NM_001002294.2:c.472G > A	0.383^b^	NP_001002294.1:p.Glu158Lys	64^†^, 98^†^, 113^†^, 114^†^, 122^†^	[37, 50]
*FMO3*	rs1736557 chr1:g.171080080G > A NM_001002294.2:c.769G > A	0.080^b^	NP_001002294.1:p.Val257Met	122^†^	[37, 51]
*FMO3*	rs2266780 chr1:g.171083242A > G NM_001002294.2:c.923A > G	0.153^b^	NP_001002294.1:p.Glu308Gly	98^†^	[50]
*PYROXD2*	rs7072216^c^ chr10:g.100156853 T > C NM_032709.2:c.625 + 249A > G	0.4012^d^	Intronic	52^†^, 56^†^, 35^§^, 64^§^, 113^§^	[21]

^a^Zygosity of samples: ^†^heterozygous; ^§^homozygous

^b^Frequency from ExAC database (http://exac.broadinstitute.org/)

^c^This intronic variant is not seen in the exome data. We selected it based on literature search and genotyped it in the ten study samples

^d^Frequency from the 1000 Genomes Project (http://www.1000genomes.org/)

**Fig. 1:**
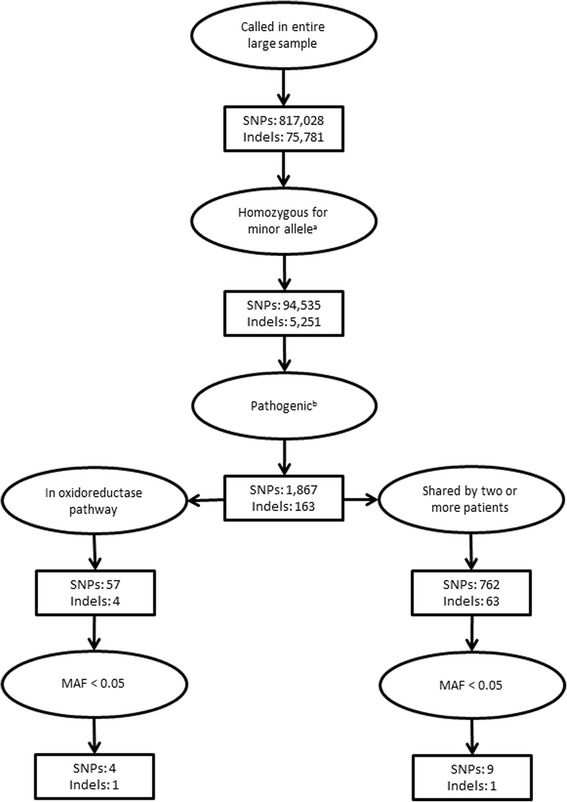
Filtering process to identify variants that could contribute to TMAU that are not in FMO3 or other genes previously and directly associated with TMA metabolism

We next looked beyond the known genes and their variants previously implicated in TMAU or TMA metabolism for variants that are rare and predicted to have reduced or abolished function. Specifically we focused on novel variants of which at least one of the ten subjects was homozygous for the alternative alleles, identifying 94,535 SNPs and 5,212 indels. The pathogenicity of these variants was predicted by algorithms implemented in either PolyPhen-2 or SIFT; 1,867 SNPs and 163 indels were predicted to be deleterious. Among these pathogenic variants, 57 SNPs and four indels were in oxidoreductase pathways different from that of the *FMO3* gene, and four of these SNPs and one indel were rare, with MAF < 0.05. Four of these five variants were found in three subjects with the lowest TMAO:TMA ratios. In addition, within other genes with no known function in TMA metabolism, two subjects shared these rare variants for nine rare putatively pathogenic SNPs and one rare putatively pathogenic indel. We depict the filtering process for the variants in Fig. 1 and we list these results in Table 4. We also conducted the gene-gene network analysis inspired by STRING results, and similar variant analysis was expanded to genes interacted with each of *FMO3* (Additional file 1: Figure S1), *PYROXD2* (Additional file 1: Figure S2) and *DMGDH* (Additional file 1: Figure S3). We found rare putatively pathogenic SNPs in multiple genes interacting with *DMGDH*, including *BHMT2* (73% identical to the homologous *BHMT* which is directly interacted with *DMGDH*), *SARDH* and *SHMT1* (Table 4). All of the subjects with these SNPs were heterozygous. We found no rare deleterious variants from genes that are currently known to interact with *FMO3* or *PYROXD2*.

**Table 4:** Novel genetic variants with potential involvement in TMAU

Variants	Chr	Pos	Gene	MAF	Subject ID^a^
SNPs in oxidoreductase pathways					
rs61733458	3	148,916,215	*CP*	0.0110	52
rs34625494	17	41,002,169	*AOC2*	0.0032	62
rs72947591	18	9,887,167	*TXNDC2*	0.0179	52
rs116368403	19	41,600,254	*CYP2A13*	0.0046	122
Indels in oxidoreductase pathways					
1 bp insertion	10	102,295,637	*HIF1AN*	0.0200	114
SNPs in DMGDH interactome					
rs58580238	5	78,378,644	*BHMT2*	0.000154	56
rs35664470	9	136,584,082	*SARDH*	0.00692	99
rs78909145	17	18,243,524	*SHMT1*	0.00701	98
SNPs shared by at least two subjects					
rs73891273	3	196,235,191	*SMCO1*	0.0445	62 & 99
rs77469804	6	110,679,450	*METTL24*	0.0262	114 & 122
rs77749341	7	149,462,317	*ZNF467*	0.0142	99 & 114
rs7091756	10	1,094,906	*IDI1*	0.0207	35 & 56
rs7956250	12	93,966,693	*SOCS2*	0.0257	99 & 122
rs55739813	15	41,803,754	*LTK*	0.0367	35 & 52
rs138735905	19	17,638,121	*FAM129C*	0.0193	64 & 98
rs114989947	22	17,265,194	*XKR3*	0.0344	99 & 113
rs41305431	X	103,495,552	*ESX1*	0.0266	113 & 114
Indels shared by at least two subjects					
1 bp insertion —	1	158,533,298	*OR6P1*	0.0100	62 & 122

rs#: reference SNP identifier (does not apply to indels). *Chr* Chromosome, *Pos* base pair position in map GRCh37/hg19, *MAF* minor allele frequency^. a^All subjects are homozygous for the minor allele

We show a summary of the number and type of gene variants detected for each subject in Table 5. Six subjects had variants in the *FMO3* gene that reduce or abolish function of the protein but four did not. However, those subjects did have rare variants predicted to be deleterious in other genes that might account for their inability to metabolize TMA. For instance, one subject (with #52) had a very low ratio of TMAO:TMA (0.13) and while she did not have any detectible variants in the *FMO3* gene, she did have two rare variants in other genes annotated as oxidoreductases (*CP* and *TXNDC2*). Similarly, another subject (#35) had no detectible *FMO3* gene variants but was homozygous for the *PYROXD2* variant that is associated with high TMA values in urine among healthy people (rs7072216).

**Table 5:** Summary of number of deleterious genetic variants by subject

ID	TMAO: TMA	Total	*FMO3*	*PYROXD2*	Oxido-reductase	DMGDH interact	Rare & shared
52	0.13	4		†	§§		§
114	0.37	7	†	§§	§		§§*
122	0.47	7	††	§	§		§§§
35	0.54	4		††			§§
99	0.58	7	§	§		†	§§§§
64	0.61	3	†	§			§
62	0.79	4		§	§		§§
113	0.79	3	†				§*
98	0.86	5	††	§		†	§
56	0.87	5	†	††		†	§

Total = number of variants in all categories. † or § denotes the presence of a relevant allele in that category. Heterozygous = †, homozygous = §, hemizygous = *. FMO3 = variants in the *FMO3* gene. PYROXD2 = variants in the *PYROXD2* gene (see Table 3). Oxidoreductase = rare variants predicted to be deleterious in genes annotated for oxidoreductase function (similar pathway as FMO3). DMGDH interact = rare variants predicted to be deleterious in genes that are predicted to interact with DMGDH, a gene linked to similar symptoms as TMAU. Rare & shared = rare variants predicted to be deleterious that are shared between two subjects studied here (see Table 4). Table is ordered by TMAO:TMA ratios, starting with the most severely impaired subject

### Sanger and Taqman sequencing compared with exome results

When comparing the results of Sanger sequencing and Taqman genotyping versus WES, 29 of the 29 of the Sanger genotypes matched and 30 out of 30 of the Taqman genotypes matched (Additional file 1: Table S3 and S4). Overall, 60 of 60 genotypes matched for a concordance rate of 100%.

## Discussion

All ten subjects studied had TMAU as defined by an impaired ability to metabolize choline during the challenge test relative to the reference values for healthy populations. Interestingly, only one of the subjects had a presumed loss-of-function variant in the *FMO3* gene (rs72549325), predicted to cause a truncated form of the *FMO3* enzyme. People who are heterozygous for this variant have reduced caffeine metabolism compared with those with alternative genotypes [40] and it may have a similar effect on TMA metabolism. We detected several other common variants in the *FMO3* gene that can impair TMA metabolism and might account, alone or in combination, for some or most of the results of the choline challenge test. As one example, subject 122 was a compound heterozygote for two common variants in the *FMO3* gene.

Two additional genes were previously but indirectly implicated in TMAU: *PYROXD2* and a *DMGDH* (dimethylglycine dehydrogenase). Common variants of the *PYROXD2* gene are associated with small increases of TMA in urine [21], and it is logical to assume that variants exist that might be more disruptive and have commensurately greater effects on TMA accumulation. However, we observed no rare disruptive variants in this study, although for the *PYROXD2* gene, many subject had the common variant identified in genome wide association studies of healthy people. This negative result for rare variants does not preclude involvement of the *PYROXD2* gene in creating TMAU-related symptoms. WES is designed to capture variants located at exons as well as near splicing sites of the human genome, and has limits in detecting genetic variants outside the exonic regions, e.g. the flanking untranslated regions as well as upstream and downstream regulatory regions. In addition, for this gene and all others studied here, there may have been loss-of-function variants we missed due to the incomplete sequencing coverage/depth of designed gene targets or limitations in evaluation of allelic function. Likewise, none of the subjects in this study showed loss-of-function *DMGDH* variants. However, we did identify rare deleterious variants in the *BHMT2*, *SARDH* and *SHMT1* genes, which directly interact with *DMGDH* in the gene network and may participate in the same pathway.

While all subjects have TMAU as evinced by the choline challenge test, none had a detectible fish-like odor during testing. All were evaluated after an overnight fast providing further credence to the role of choline restriction, albeit difficult, as a means of symptom amelioration. One of the frustrations of people with TMAU is that their odor symptoms are episodic and may not be detectible during medical visits, and because the odor has come to be known as the cardinal feature of the disease, this sometimes leads to delays in diagnosis. The results of this study illustrate the point– the choline challenge results indicated problems in TMA metabolism and all the patients had sought treatment because of body odor complaints. However, many subjects had halitosis, which is not indicative of this specific metabolic disease but is common in many populations [41], and which may be confused with “body odor” unless one performs sensory evaluation of the oral cavity and upper body as we did here. Some people persistently complain of body odor that is never detectable to others, a common form of delusion called Olfactory Reference Syndrome [42] which sometimes manifests itself with the mistaken belief that the person has TMAU [43]. With the increased ease of communication through the internet, the volume of people with Olfactory Reference Syndrome can overwhelm the small number of clinics and researchers who study TMAU. Thus, the choline challenge test is useful in deciding if the patient has TMAU because the fish-like odor is not present consistently.

Malodor may not be the only medically and psychologically important outcome of TMAU. Higher levels of TMAO, the oxidized form of TMA, are associated with increased incidence of atherosclerosis [44–46]. Thus, it would be of interest to learn whether people with TMAU have a lower incidence of this disease, e.g., to determine whether people who produce less TMAO than others may have a lower risk of cardiovascular disease. Odor production may have an inverse relationship with cardiovascular health benefits.

Low-choline diets are recommendation for TMAU symptom management, but susceptible people can develop organ damage if the diet is too low in choline [47]. Some people are susceptible because of their genotype and others may be especially vulnerable because of their particular gut microbiota which in some people can over-convert choline into TMA [2]. If these particular bacteria are present in high levels, they can deprive the person of choline needed for other metabolic pathways [48]. To circumvent this problem, investigators have suggested that probiotics be used to treat TMAU, e.g., by converting TMA in the gut to an odorless compound, although technical challenges currently preclude immediate testing of this suggestion [49].

## Conclusions

In summary, WES of ten subjects revealed genetic variants that could potentially cause TMAU. Our results suggest there could be multiple impairments of genes and their protein products along this metabolic pathway can cause accumulation of clinically significant amounts of TMA. These genes provide a new avenue to pursue when understanding the cause underlying body odor complaints. Finally, our results further suggest that the choline challenge test may be the most direct way to reveal putative deficits in oxidoreductase pathways, and larger sample size coupled with more comprehensive genetic methods such as whole genome sequencing will facilitate the discovery of more TMAU causative/related variants, either in the known genes e.g. FMO3 or in novel genes implicated by gene-gene interactions.

## Additional file

Additional file 1: Table S1. Variant distribution for exome sequencing. Table S2. Known pathogenic alleles in *FMO3* and *PYROXD2* not detected in these subjects. Table S3: Exome versus Sanger sequencing. Table S4: Exome versus Taqman genotypes. Figure S1. Gene-gene interaction network for *FMO3* generated by STRING. Figure S2. Gene-gene interaction network for *PYROXD2* generated by STRING. Figure S3. Gene-gene interaction network for *DMGDH* generated by STRING. Supplemental references. (DOC 784 kb)

## Abbreviations

SNP: Single nucleotide polymorphism
TMA: Trimethylamine
TMAO: Trimethylamine N-oxide
TMAU: Trimethylaminuria
