# Supplementary material for: Genetic analysis of impaired trimethylamine metabolism using whole exome sequencing
Source: BMC Med Genet. 2017 Feb 15;18:11. doi: 10.1186/s12881-017-0369-8 (PMC5310055; doi:10.1186/s12881-017-0369-8)
Supplement: Additional file 1: — Table S1. Variant distribution for exome sequencing. Table S2. Known pathogenic alleles in FMO3 and PYROXD2 not detected in these subjects. Table S3: Exome versus Sanger sequencing. Table S4: Exome versus Taqman genotypes. Figure S1. Gene-gene interaction network for FMO3 generated by STRING. Figure S2. Gene-gene interaction network for PYROXD2 generated by STRING. Figure S3. Gene-gene interaction network for DMGDH generated by STRING. Supplemental references. (DOC 784 kb) [file 12881_2017_369_MOESM1_ESM.doc]

**Genetic Analysis of Impaired Trimethylamine Metabolism Using Whole Exome Sequencing**

Yiran Guo, Liang-Dar Hwang, Jiankang Li, Jason Eades, Chung Wen Yu, Corrine Mansfield, Alexis Burdick-Will, Xiao Chang, Yulan Chen, Fujiko F. Duke, Jianguo Zhang, Steven Fakharzadeh, Paul Fennessey, Brendan J. Keating, Hui Jiang, Hakon Hakonarson, Danielle R. Reed, and George Preti

**Supplementary material**

**Supplemental Tables 1 - 4**

**Supplemental Figures 1 - 3**

**Supplemental References**

**Table S1.** Variant distribution for exome sequencing.

| **Subject ID** | **SNP** | | | |  | **Indel** | | | |
| --- | --- | --- | --- | --- | --- | --- | --- | --- | --- |
|  | **+/+** | **+/-** | **-/-** | **No call** |  | **+/+** | **+/-** | **-/-** | **No call** |
| 35 | 567,764 | 34,537 | 27,212 | 187,515 |  | 38,591 | 6,989 | 4,894 | 25,307 |
| 52 | 583,034 | 36,366 | 28,829 | 168,799 |  | 40,205 | 7,376 | 5,318 | 22,882 |
| 56 | 607,416 | 38,246 | 35,156 | 136,210 |  | 41,958 | 7,242 | 6,568 | 20,013 |
| 62 | 596,604 | 46,700 | 35,269 | 138,455 |  | 41,362 | 7,779 | 6,584 | 20,056 |
| 64 | 608,776 | 37,954 | 35,050 | 135,248 |  | 41,986 | 7,212 | 6,395 | 20,188 |
| 98 | 564,552 | 45,815 | 28,871 | 177,790 |  | 38,716 | 7,989 | 5,373 | 23,703 |
| 99 | 570,418 | 46,512 | 31,408 | 168,690 |  | 39,017 | 8,032 | 5,721 | 23,011 |
| 113 | 618,966 | 37,954 | 37,398 | 122,710 |  | 43,002 | 7,327 | 6,803 | 18,649 |
| 114 | 571,751 | 45,318 | 31,293 | 168,666 |  | 38,872 | 7,682 | 5,660 | 23,567 |
| 122 | 593,092 | 47,600 | 35,506 | 140,830 |  | 40,688 | 8,118 | 6,501 | 20,474 |

SNP: single-nucleotide polymorphism. indel: insertion/deletion. +: reference alleles. –: alternative alleles. No call: genotype could not be assigned for a particular variant site that is polymorphic in at least one individual among a larger sample pool (n=669).

**Table S2.** Known pathogenic alleles in *FMO3* and *PYROXD2* not detected in these subjects.

| **Gene** | **Variants (hg19)** | **MAF^a^** | **Amino acid change** | **Ref^b^** |
| --- | --- | --- | --- | --- |
| *FMO3* | rs72549320; chr1:g.171061893G>A; NM_001002294.2:c.94G>A | — | NP_001002294.1:p.Glu32Lys |  |
| *FMO3* | rs72549321; chr1:g.171072947G>A; NM_001002294.2:c.154G>A | — | NP_001002294.1:p.Ala52Thr |  |
| *FMO3* | rs144935285; chr1:g.171072965G>A; NM_001002294.2:c.172G>A | 4.283e-04 | NP_001002294.1:p.Val58Ile |  |
| *FMO3* | rs72549322; chr1:g.171072975A>G; NM_001002294.2:c.182A>G | 1.647e-05 | NP_001002294.1:p.Asn61Ser |  |
| *FMO3* | rs72549323; chr1:g.171072991G>T; NM_001002294.2:c.198G>T | 8.237e-06 | NP_001002294.1:p.Met66Ile |  |
| *FMO3* | rs72549324; chr1:g.171073038T>C; NM_001002294.2:c.245T>C | 9.885e-05 | NP_001002294.1:p.Met82Thr |  |
| *FMO3* | rs186763441; chr1:g.171076835A>G; NM_001002294.2:c.341A>G | 6.590e-04 | NP_001002294.1:p.Asn114Ser |  |
| *FMO3* | rs12072582; chr1:g.171076888G>C; NM_001002294.2:c.394G>C/  chr1:g.171076888G>T; NM_001002294.2:c.394G>T | 3.509e-03/ 8.237e-06 | NP_001002294.1:p.Asp132His/  NP_001002294.1:p.Asp132Tyr |  |
| *FMO3* | rs72549326; chr1:g.171076952C>T; NM_001002294.2:c.458C>T | 9.884e-04 | NP_001002294.1:p.Pro153Leu |  |
| *FMO3* | rs75904274; chr1:g.171077274G>T; NM_001002294.2:c.539G>T | 1.300e-02 | NP_001002294.1:p.Gly180Val |  |
| *FMO3* | rs3832024; chr1:g.171077326_171077327delTG;  NM_001002294.2:c.591_592delTG | — | NP_001002294.1:p.Cys197Terfs |  |
| *FMO3* | rs28363549; chr1:g.171077348C>T; NM_001002294.2:c.613C>T | 2.142e-04 | NP_001002294.1:p.Arg205Cys |  |
| *FMO3* | rs61753344; chr1:g.171083232G>T; NM_001002294.2:c.913G>T | 3.048e-04 | NP_001002294.1:p.Glu305Ter |  |
| *FMO3* | rs72549330; chr1:g.171083259G>A; NM_001002294.2:c.940G>A  chr1:g.171083259G>T; NM_001002294.2:c.940G>T | 8.237e-06/  — | NP_001002294.1:p.Glu314Lys/  NP_001002294.1:p.Glu314Ter |  |
| *FMO3* | rs28363581; chr1:g.171083398T>C; NM_001002294.2:c.1079T>C | 3.871e-04 | NP_001002294.1:p.Leu360Pro |  |
| *FMO3* | rs2066532; chr1:g.171083403G>C; NM_001002294.2:c.1084G>C | 1.384e-03 | NP_001002294.1:p.Glu362Gln |  |
| *FMO3* | rs72549331; chr1:g.171083479G>A; NM_001002294.2:c.1160G>A/  chr1:g.171083479G>T; NM_001002294.2:c.1160G>T | 1.071e-04/  — | NP_001002294.1:p.Arg387His/  NP_001002294.1:p.Arg387Leu |  |
| *FMO3* | rs61757397; chr1:g.171086245G>T; NM_001002294.2:c.1262G>T | — | NP_001002294.1:p.Gly421Val |  |
| *FMO3* | rs72549332; chr1:g.171086285G>A; NM_001002294.2:c.1302G>A | 6.589e-05 | NP_001002294.1:p.Met434Ile |  |
| *FMO3* | rs72549333; chr1:g.171086407G>A; NM_001002294.2:c.1424G>A | 5.766e-05 | NP_001002294.1:p.Gly475Asp |  |
| *FMO3* | rs72549334; chr1:g.171086457C>T; NM_001002294.2:c.1474C>T | 1.153e-04 | NP_001002294.1:p.Arg492Trp |  |
| *FMO3* | rs72549335; chr1:g.171086490G>A; NM_001002294.2:c.1507G>A | 8.237e-06 | NP_001002294.1:p.Gly503Arg |  |
| *PYROXD2* | rs2147896; chr10:g.100148176A>G; NM_032709.2:c.1382T>C | 4.690e-01 | NP_116098.2:p.Met461Thr |  |

Minor alleles of all SNPs for the *FMO3* are associated with increased risk of TMAU. MAF=minor allele frequency.

^a^Frequencies in the ExAC database of ~60,000 samples. ^c^References are listed at the end of this document.

**Table S3**: Exome versus Sanger sequencing

| **Subject ID** | **Variant** | **Exome** | **Sanger** |
| --- | --- | --- | --- |
| 35 | rs1736557 | G:G | G:G |
| 35 | rs2066532 | G:G | G:G |
| 35 | rs61753344 | G:G | G:G |
| 35 | rs28363581 | T:T | T:T |
| 62 | rs1736557 | G:G | G:G |
| 62 | rs12072582 | G:G | G:G |
| 62 | rs2266782 | G:G | G:G |
| 64 | rs2266782 | A:G | A:G |
| 64 | rs1736557 | G:G | G:G |
| 64 | rs12072582 | G:G | G:G |
| 99 | rs1736557 | G:G | G:G |
| 99 | rs12072582 | G:G | G:G |
| 99 | rs2266782 | G:G | G:G |
| 113 | rs2266780 | A:A | A:A |
| 113 | rs2266782 | A:G | A:G |
| 113 | rs12072582 | G:G | G:G |
| 113 | rs2066532 | G:G | G:G |
| 113 | rs61753344 | G:G | G:G |
| 113 | rs28363581 | T:T | T:T |
| 114 | rs2266782 | A:G | A:G |
| 114 | rs1736557 | G:G | G:G |
| 114 | rs12072582 | G:G | G:G |
| 122 | rs2266780 | A:A | A:A |
| 122 | rs2266782 | A:G | A:G |
| 122 | rs1736557 | A:G | A:G |
| 122 | rs12072582 | G:G | G:G |
| 122 | rs2066532 | G:G | G:G |
| 122 | rs61753344 | G:G | G:G |
| 122 | rs28363581 | T:T | T:T |

#All genotypes from Sanger sequencing agreed with the Exome genotype. *rs7072216* is from the *PYROXD2* gene; all others are from the *FMO3* gene. We ordered this table by subject identification number.

**Table S4**: Exome versus Taqman genotypes

| **Subject ID** | **Variant** | **Exome** | **Taqman** |
| --- | --- | --- | --- |
| 35 | rs2266780 | A:A | A:A |
| 35 | rs2266782 | G:G | G:G |
| 35 | rs2066532 | T:T | T:T |
| 52 | rs2266780 | A:A | A:A |
| 52 | rs2066532 | C:T | C:T |
| 52 | rs2266782 | G:G | G:G |
| 56 | rs2266780 | A:A | A:A |
| 56 | rs2066532 | C:T | C:T |
| 56 | rs2266782 | G:G | G:G |
| 62 | rs2266780 | A:A | A:A |
| 62 | rs2066532 | C:C | C:C |
| 62 | rs2266782 | G:G | G:G |
| 64 | rs2266780 | A:A | A:A |
| 64 | rs2266782 | A:G | A:G |
| 64 | rs2066532 | T:T | T:T |
| 98 | rs2266782 | A:G | A:G |
| 98 | rs2266780 | A:G | A:G |
| 98 | rs2066532 | C:C | C:C |
| 99 | rs2266780 | A:A | A:A |
| 99 | rs2066532 | C:C | C:C |
| 99 | rs2266782 | G:G | G:G |
| 113 | rs2266780 | A:A | A:A |
| 113 | rs2266782 | A:G | A:G |
| 113 | rs2066532 | T:T | T:T |
| 114 | rs2266780 | A:A | A:A |
| 114 | rs2266782 | A:G | A:G |
| 114 | rs2066532 | C:C | C:C |
| 122 | rs2266780 | A:A | A:A |
| 122 | rs2266782 | A:G | A:G |
| 122 | rs2066532 | C:C | C:C |

See Supplemental Table 2 caption for details.

**Figure S1. Gene-gene interaction network for *FMO3* generated by STRING.**


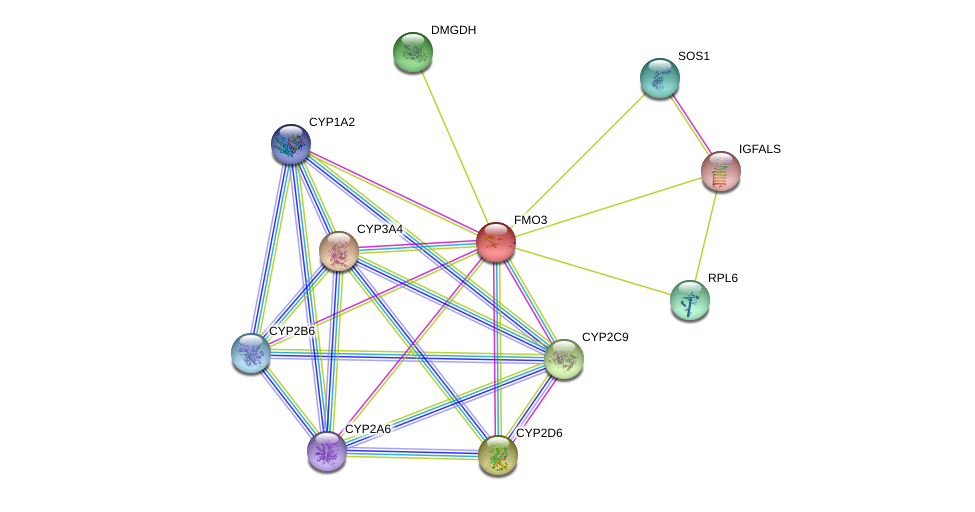


**Figure S2. Gene-gene interaction network for *PYROXD2* generated by STRING.**


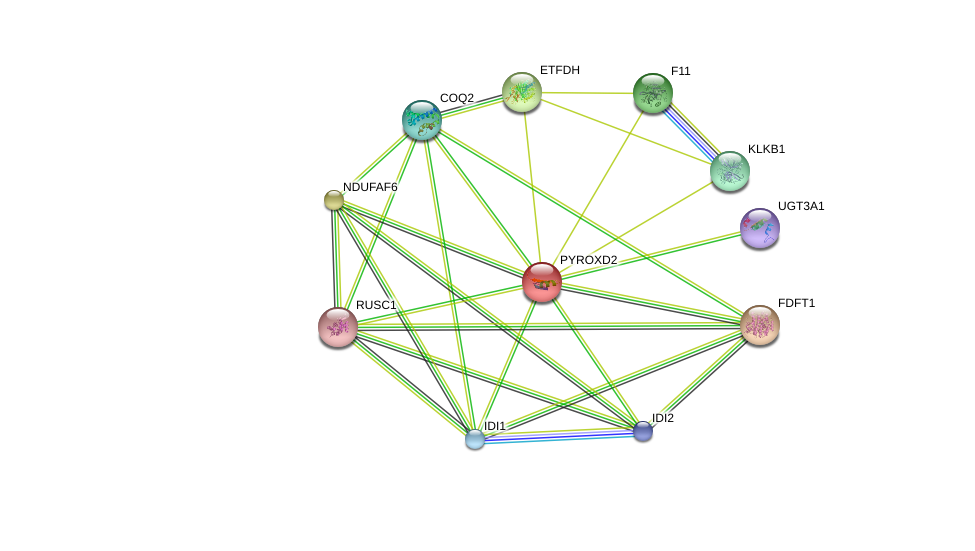


**Figure S3. Gene-gene interaction network for *DMGDH* generated by STRING.**


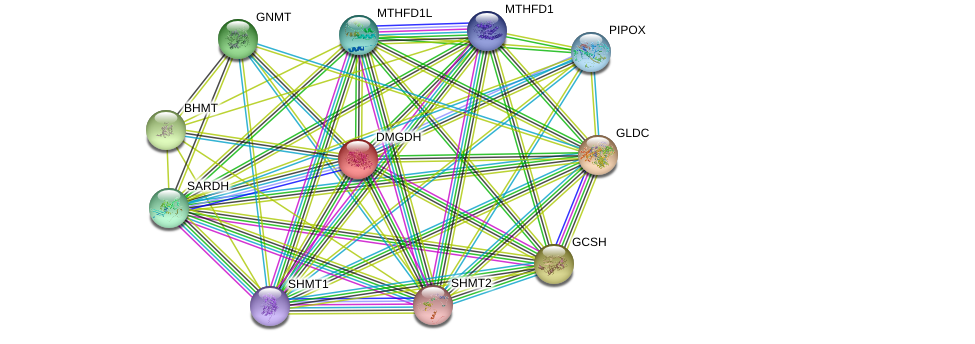


**Supplemental** References

1. Zhang J, Tran Q, Lattard V, Cashman JR: **Deleterious mutations in the flavin-containing monooxygenase 3 (FMO3) gene causing trimethylaminuria.** *Pharmacogenetics* 2003, **13:**495-500.

2. Akerman BR, Lemass H, Chow LM, Lambert DM, Greenberg C, Bibeau C, Mamer OA, Treacy EP: **Trimethylaminuria is caused by mutations of the FMO3 gene in a North American cohort.** *Mol Genet Metab* 1999, **68:**24-31.

3. Kubota M, Nakamoto Y, Nakayama K, Ujjin P, Satarug S, Mushiroda T, Yokoi T, Funayama M, Kamataki T: **A mutation in the flavin-containing monooxygenase 3 gene and its effects on catalytic activity for N-oxidation of trimethylamine in vitro.** *Drug Metab Pharmacokinet* 2002, **17:**207-213.

4. Dolphin CT, Janmohamed A, Smith RL, Shephard EA, Phillips IR: **Compound heterozygosity for missense mutations in the flavin-containing monooxygenase 3 (FM03) gene in patients with fish-odour syndrome.** *Pharmacogenetics* 2000, **10:**799-807.

5. Treacy EP, Akerman BR, Chow LM, Youil R, Bibeau C, Lin J, Bruce AG, Knight M, Danks DM, Cashman JR, Forrest SM: **Mutations of the flavin-containing monooxygenase gene (FMO3) cause trimethylaminuria, a defect in detoxication.** *Hum Mol Genet* 1998, **7:**839-845.

6. Akerman BR, Forrest S, Chow L, Youil R, Knight M, Treacy EP: **Two novel mutations of the FMO3 gene in a proband with trimethylaminuria.** *Hum Mutat* 1999, **13:**376-379.

7. Murphy HC, Dolphin CT, Janmohamed A, Holmes HC, Michelakakis H, Shephard EA, Chalmers RA, Phillips IR, Iles RA: **A novel mutation in the flavin-containing monooxygenase 3 gene, FM03, that causes fish-odour syndrome: activity of the mutant enzyme assessed by proton NMR spectroscopy.** *Pharmacogenetics* 2000, **10:**439-451.

8. Shimizu M, Tomioka S, Murayama N, Yamazaki H: **Missense and nonsense mutations of the flavin-containing monooxygenase 3 gene in a Japanese cohort.** *Drug Metab Pharmacokinet* 2007, **22:**61-64.

9. Yamazaki H, Shimizu M: **Genetic polymorphism of the flavin-containing monooxygenase 3 (FMO3) associated with trimethylaminuria (fish odor syndrome): observations from Japanese patients.** *Curr Drug Metab* 2007, **8:**487-491.

10. Furnes B, Feng J, Sommer SS, Schlenk D: **Identification of novel variants of the flavin-containing monooxygenase gene family in African Americans.** *Drug Metab Dispos* 2003, **31:**187-193.

11. Lattard V, Zhang J, Tran Q, Furnes B, Schlenk D, Cashman JR: **Two new polymorphisms of the FMO3 gene in Caucasian and African-American populations: comparative genetic and functional studies.** *Drug Metab Dispos* 2003, **31:**854-860.

12. Zschocke J, Kohlmueller D, Quak E, Meissner T, Hoffmann GF, Mayatepek E: **Mild trimethylaminuria caused by common variants in FMO3 gene.** *Lancet* 1999, **354:**834-835.

13. Teresa E, Lonardo F, Fiumara A, Lombardi C, Russo P, Zuppi C, Scarano G, Musumeci S, Gianfrancesco F: **A spectrum of molecular variation in a cohort of Italian families with trimethylaminuria: identification of three novel mutations of the FM03 gene.** *Mol Genet Metab* 2006, **88:**192-195.

14. Park CS, Chung WG, Kang JH, Roh HK, Lee KH, Cha YN: **Phenotyping of flavin-containing monooxygenase using caffeine metabolism and genotyping of FMO3 gene in a Korean population.** *Pharmacogenetics* 1999, **9:**155-164.

15. Yamazaki H, Fujita H, Gunji T, Zhang J, Kamataki T, Cashman JR, Shimizu M: **Stop codon mutations in the flavin-containing monooxygenase 3 (FMO3) gene responsible for trimethylaminuria in a Japanese population.** *Mol Genet Metab* 2007, **90:**58-63.

16. Shimizu M, Kobayashi Y, Hayashi S, Aoki Y, Yamazaki H: **Variants in the flavin-containing monooxygenase 3 (FMO3) gene responsible for trimethylaminuria in a Japanese population.** *Mol Genet Metab* 2012, **107:**330-334.

17. Basarab T, Ashton GH, Menage HP, McGrath JA: **Sequence variations in the flavin-containing mono-oxygenase 3 gene (FMO3) in fish odour syndrome.** *Br J Dermatol* 1999, **140:**164-167.

18. D'Angelo R, Esposito T, Calabro M, Rinaldi C, Robledo R, Varriale B, Sidoti A: **FMO3 allelic variants in Sicilian and Sardinian populations: trimethylaminuria and absence of fish-like body odor.** *Gene* 2013, **515:**410-415.

19. Rueedi R, Ledda M, Nicholls AW, Salek RM, Marques-Vidal P, Morya E, Sameshima K, Montoliu I, Da Silva L, Collino S, et al: **Genome-wide association study of metabolic traits reveals novel gene-metabolite-disease links.** *PLoS Genet* 2014, **10:**e1004132.
